# Comparing the euploidy rate between the progestin-primed and gonadotrophin-releasing hormone antagonist protocols in preimplantation genetic testing for aneuploidy: a randomized controlled trial

**DOI:** 10.1093/hropen/hoag081

**Published:** 2026-09-15

**Authors:** He Li, Min Yu, Wenbi Zhang, Junling Chen, Hua Chen, Xiang Lu, Lu Li, Ernest Hung Yu Ng, Xiaoxi Sun

**Affiliations:** Department of Obstetrics and Gynaecology, School of Clinical Medicine, Li Ka Shing Faculty of Medicine, The University of Hong Kong, Hong Kong, China; Shanghai Ji Ai Genetics and IVF Institute, Obstetrics and Gynecology Hospital, Fudan University, Shanghai, China; Shanghai Ji Ai Genetics and IVF Institute, Obstetrics and Gynecology Hospital, Fudan University, Shanghai, China; Department of Obstetrics and Gynecology, Ruijin Hospital, Shanghai Jiao Tong University School of Medicine, Shanghai, China; Department of Reproductive Medicine, Ren Ji Hospital, Shanghai Jiao Tong University School of Medicine, Shanghai, China; Shanghai Ji Ai Genetics and IVF Institute, Obstetrics and Gynecology Hospital, Fudan University, Shanghai, China; Shanghai Ji Ai Genetics and IVF Institute, Obstetrics and Gynecology Hospital, Fudan University, Shanghai, China; Shanghai Ji Ai Genetics and IVF Institute, Obstetrics and Gynecology Hospital, Fudan University, Shanghai, China; Department of Obstetrics and Gynaecology, School of Clinical Medicine, Li Ka Shing Faculty of Medicine, The University of Hong Kong, Hong Kong, China; Shanghai Ji Ai Genetics and IVF Institute, Obstetrics and Gynecology Hospital, Fudan University, Shanghai, China; Key Laboratory of Female Reproductive Endocrine Related Diseases, Obstetrics and Gynecology Hospital, Fudan University, Shanghai, China; Obstetrics and Gynecology Hospital, Fudan University, Shanghai, China

**Keywords:** antagonist protocol, progestin-primed ovarian stimulation, euploidy rate, preimplantation genetic testing for aneuploidy, IVF

## Abstract

**STUDY QUESTION:**

In patients undergoing preimplantation genetic testing for aneuploidy (PGT-A), does the progestin-primed ovarian stimulation (PPOS) protocol result in a euploidy rate of blastocysts per patient comparable to that of the GnRH antagonist protocol?

**SUMMARY ANSWER:**

The euploidy rate of blastocysts per patient is comparable for patients using PPOS and GnRH antagonist protocols.

**WHAT IS KNOWN ALREADY:**

Progestin is effective in blocking the pituitary LH surge during ovarian stimulation, and the PPOS protocol is simpler and cheaper. However, published studies comparing euploidy rates between the two protocols have yielded contradictory results.

**STUDY DESIGN, SIZE, DURATION:**

This was an open-label, randomized controlled trial of 400 women recruited between June 2020 and August 2024. Eligible women were randomly assigned in a ratio of 1:1 to the PPOS or GnRH antagonist group using an online randomization programme and sealed opaque envelopes. The primary outcome was the euploidy rate of blastocysts per patient.

**PARTICIPANTS/MATERIALS, SETTING, METHODS:**

Women aged <43 years undergoing PGT-A cycles were randomly assigned to either the PPOS group (n = 200) or antagonist group (n = 200). PGT-A was indicated for advanced maternal age, recurrent pregnancy loss, repeated implantation failure, or history of foetal aneuploidy in previous pregnancies. PGT-A was performed using the next-generation sequencing technology.

**MAIN RESULTS AND THE ROLE OF CHANCE:**

The PPOS and antagonist groups were similar in demographic characteristics and the numbers of oocytes obtained/fertilized, cleavage-stage embryos, and developed blastocysts. The PPOS group had a significantly lower total gonadotrophin doses and significantly higher serum oestradiol levels on the ovulation trigger day when compared to the GnRH antagonist group. No statistically significant difference was observed in the euploidy rate of blastocysts between the PPOS and antagonist groups (40.0 (0–66.7)% versus 44.4 (25.0–71.4)%, *P* = 0.192). The number of euploid blastocysts per patient was comparable for the PPOS and antagonist groups (1 (1–2) versus 1 (1–2.5), *P* = 0.47). There were 136 women in the PPOS group and 152 women in the antagonist group who had their first frozen embryo transfer after PGT-A. Both groups showed comparable clinical pregnancy, ongoing pregnancy, miscarriage, ectopic pregnancy, and live birth rates after the first frozen embryo transfer.

**LIMITATIONS, REASONS FOR CAUTION:**

The participants and researchers were not blinded to the treatment allocation. The sample size was powered only to detect a 10% euploidy rate difference; smaller differences cannot be excluded. Not all women completed their first transfer cycle, and outcomes from a single cycle do not represent cumulative live birth rates. Results are based solely on euploid embryo transfers, limiting generalizability to mosaic embryos. Generalization to other IVF populations or to the use of other progestins is also limited.

**WIDER IMPLICATION OF THE FINDINGS:**

The findings of the study support the use of PPOS for patients undergoing a freeze-all IVF cycle, whether for PGT-A or oocyte preservation.

**FUNDING:**

This study was supported by Shanghai Oriental Talent Award (QNWS2024045), the National Natural Science Foundation of China (82171644), and Shanghai Shen Kang Hospital Development Center Municipal Hospital New Frontier Technology Joint Project (SHDC12017105).

**DISCLOSURES:**

The authors report no conflicts of interest.

**TRIAL REGISTRATION NUMBER:**

NCT04414748.

**TRIAL REGISTRATION DATE:**

4 June 2020.

**DATE OF FIRST PATIENT’S ENROLLMENT:**

10 June 2020.

WHAT DOES THIS MEAN FOR PATIENTS? During ovarian stimulation for IVF, follicle stimulating hormone (FSH) is given to develop multiple follicles, while a gonadotrophin-releasing hormone (GnRH) antagonist is commonly injected to prevent early ovulation. Another approach, called progestin-primed ovarian stimulation (PPOS), uses an oral drug (progestin) to achieve the same goal, but it is simpler, user friendly, and less expensive. However, there is still some concern that the PPOS protocol might lead to a higher number of chromosomal abnormalities of embryos. In this study, we compared the two protocols (GnRH antagonist versus PPOS) in women who had their embryos tested for chromosomal abnormalities before transfer to their uterus. We found that the two patient groups had similar percentages of embryos with a normal number of chromosomes, which is important for a successful pregnancy. We also found that pregnancy rates and birth rates of live babies were similar between the two groups. These results suggest that PPOS is a good alternative for patients, particularly those who are freezing all embryos for later use, whether for preimplantation genetic testing for aneuploidy or fertility preservation.

## Introduction

Progestin can inhibit the pituitary LH surge during ovarian stimulation, and various studies have shown that progestin-primed ovarian stimulation (PPOS) is effective in blocking the LH surge in IVF (Massin, 2017). More and more centres are using PPOS because this regimen is simpler and cheaper than the traditional GnRH antagonist protocol. However, due to the negative impact of progestin on the endometrium, fresh embryo transfer is not feasible, and all embryos are electively frozen. Combined with the improvement in embryo cryopreservation techniques, the PPOS protocol may have broader applications in women who freeze all embryos for various reasons, such as undergoing preimplantation genetic testing, fertility preservation, or prevention of ovarian hyperstimulation syndrome.

The effectiveness and safety of the PPOS protocol have been widely investigated in recent years. Meta-analyses have shown that the PPOS and antagonist protocols result in comparable outcomes, including the number of mature oocytes, blastocyst formation rate, number of good-quality embryos, implantation rate, clinical pregnancy rate, and live birth rate (Alexandru *et al.*, 2020; Zolfaroli *et al.*, 2020; Ata *et al.*, 2021; Cui *et al.*, 2021; Guan *et al.*, 2021). But some studies have demonstrated contradictory results. A retrospective study showed that among patients aged over 38 years, those treated with the PPOS protocol had lower rates of mature oocyte retrieval, blastocyst formation, and euploid blastocysts than those receiving the antagonist protocol (Pai *et al.*, 2023). A randomized trial conducted in an oocyte donation setting found comparable numbers of mature oocytes between the PPOS and antagonist protocols. However, recipients of oocytes from donors treated with PPOS had lower ongoing pregnancy and live birth rates (Beguería *et al.*, 2019). It should be noted that the oocyte recipients in this trial were not randomized. Therefore, the effect of the progestin used in IVF on pregnancy outcomes remains controversial.

The potential impact of elevated progesterone levels on oocyte and embryo quality has been widely studied, although the findings across the literature remain inconsistent. In a retrospective cohort analysis of 4236 IVF cycles, Huang *et al.* categorized patients into five groups according to serum progesterone levels on the day of HCG administration: ≤1.00, 1.00–1.50, 1.50–2.00, 2.00–2.50, and >2.50 ng/ml. The study focused on the rate of top-quality embryos, defined as the number of top-quality embryos per two-pronuclei zygote. The results indicated a significant reduction in top-quality embryo rates among patients with progesterone levels exceeding 2 ng/ml (Huang *et al.*, 2016). Several clinical studies support the notion that serum progesterone levels above 1.5 ng/ml on the day of HCG administration adversely affect embryo quality (Vanni *et al.*, 2017; Racca *et al.*, 2018), although other reports have found no such association (Ubaldi *et al.*, 1995; Melo *et al.*, 2006; Xu *et al.*, 2012; Racca *et al.*, 2020; Pardiñas *et al.*, 2021). Given that the PPOS protocol involves ovarian stimulation under high progesterone conditions, it may similarly compromise euploidy rates, potentially leading to reduced live birth outcomes. However, a recent randomized study reported similar euploidy rates per group in patients receiving PPOS or GnRH antagonist for preimplantation genetic testing for aneuploidy (PGT-A) cycles (Wang *et al.*, 2025).

Thus, we designed this randomized controlled study to compare the euploidy rate of blastocysts following the PPOS protocol and the GnRH antagonist protocol in women undergoing PGT-A. The euploidy rate, as a critical indicator of embryo quality, can serve as a basis for assessing the effectiveness and safety of the PPOS protocol.

## Materials and methods

### Ethical approval

The study was approved by the ethics committee of assisted reproductive medicine in Shanghai JiAi Genetics and IVF Institute (JIAI E2020-03) and was conducted in accordance with the Declaration of Helsinki and good clinical practice guidelines. It was registered in ClinicalTrials.gov with an identifier number NCT04414748. All participants provided written informed consent to join the study after extensive counselling.

### Study design and participants

This randomized study was carried out at the Shanghai Ji Ai Genetics & IVF Institute from June 2020 through August 2024. The study protocol was published (Li *et al.*, 2024). Women undergoing PGT-A treatment were screened at the assisted reproduction clinics for eligibility. Patients were eligible if they fulfilled the following inclusion criteria: (i) age of women 20–43 years at the time of ovarian stimulation for IVF; (ii) antral follicle count ≥5 on Days 2–5 of the period; (iii) PGT-A indicated for advanced maternal age (≥38 years), recurrent pregnancy loss (≥2 consecutive pregnancy loss), repeated implantation failure (≥4 embryos or ≥2 blastocysts replaced without success), or history of foetal aneuploidy in previous pregnancies. Women were excluded if they had a functional ovarian cyst with serum oestradiol level >100 pg/ml on Day 2–3 of the period; were using donor oocytes; or had hydrosalpinx or endometrial polyps that were not surgically treated.

### Randomization and masking

Randomization was carried out on the day of starting ovarian stimulation by a project nurse who was not involved in the recruitment and clinical care of the women. The randomization list was generated using an online randomization programme (www.randomization.com) and then put in opaque envelopes that were opened consecutively. Recruited women were randomly assigned to the PPOS group or antagonist group. It was an open-label study for women and doctors, but the embryologists who evaluated the oocytes/embryos and the laboratory staff performing PGT-A were blinded to the treatment allocation.

### Interventions

On Day 2–3, transvaginal ultrasound was performed for the antral follicle count, and blood was taken for serum oestradiol, progesterone, and FSH levels. Recruited women were randomly assigned on the same day according to the randomization list to one of the two groups: (i) the GnRH antagonist group where ovarian stimulation was started according to the standard protocol described below if there were no ovarian cysts or follicles >10 mm in diameter, and serum oestradiol level was <100 pg/ml, and women received an antagonist (Cetrorelix 0.25 mg/day; Merck-Serono, Geneva, Switzerland) once subcutaneously daily from Day 6 of ovarian stimulation until the day of the ovulation trigger; and (ii) the PPOS group, where women received dydrogesterone (Duphaston, 20 mg/day, Abbott Biologicals B.V., Olst, the Netherlands) from the first day of ovarian stimulation until the day of the ovulation trigger.

Gonadotrophin (human menopausal gonadotrophin, Lizhu Pharmaceutical Trading Co., Zhuhai, China, or recombinant FSH, Gonal F, Merck-Serono) injections (225–300 IU/day) were started according to antral follicle count. On the sixth day of gonadotrophin injection, transvaginal ultrasound was performed, and serum LH, oestradiol, and progesterone levels were measured to monitor follicle growth. Gonadotrophin dosage was adjusted according to the ovarian response, and subsequently such monitoring was performed either every 2 or 3 days. Triggering of the final oocyte maturation was given when at least three follicles reach >17 mm in diameter. HCG (2000 IU, Lizhu Pharmaceutical Trading Co.) and GnRH agonist (decapeptyl 0.2 mg, Ferring Pharmaceuticals, Hoofddorp, the Netherlands) were given for triggering of final maturation. Blood was checked for serum oestradiol and progesterone levels on the day of the trigger. Oocyte retrieval was performed 36 h after the trigger under transvaginal ultrasound guidance.

### ICSI, embryo culture, and PGT-A

Approximately 4 h after oocyte retrieval, oocytes were fertilized using ICSI and incubated in fertilization media (G-IVF™ PLUS; Vitrolife, Göteborg, Sweden) with the standard protocols in the centres. Normal fertilization was confirmed by the presence of two pronuclei at 16–18 h after micro-injection. Zygotes were subsequently cultured in cleavage medium (G-1™ PLUS; Vitrolife). On Day 3 post-fertilization, assisted hatching was performed by creating an 18-µm opening in the zona pellucida of all embryos using a non-contact laser system (LYKOS; Hamilton Thorne, Beverly, MA, USA). Embryos were then transferred to blastocyst medium (G-2™ PLUS; Vitrolife) and cultured until the blastocyst stage, with blastocyst formation assessed on Days 5 and 6. Blastocysts were graded according to the Gardner and Schoolcraft classification (Gardner *et al.*, 2002) and were considered eligible for cryopreservation and biopsy on Day 5 or 6 after retrieval if they qualified as full (B3) or expanded (B4–5) blastocysts with a Type A–C inner cell mass and/or a Type A–C trophectoderm (TE), but blastocysts with a Type C inner cell mass and a Type C TE were not cryopreserved or biopsied.

Blastocyst biopsy was performed on the day of blastocyst formation. The procedure was carried out in buffered handling medium (G-MOPS™; Vitrolife). The blastocyst was stabilized using a holding pipette, and 5–10 TE cells were herniated and aspirated into a biopsy pipette. The herniated TE cells were then separated from the blastocyst using a combination of laser pulses (LYKOS; Hamilton Thorne) and a mechanical flick method. The biopsied cells were washed in G-MOPS™ medium, transferred into PCR tubes containing phosphate-buffered saline (PBS), and immediately sent to our genetic laboratory for analysis using the next-generation sequencing (Illumina MiSeq platform; Illumina, Inc., San Diego, CA, USA) platform for comprehensive chromosome analysis. The resolution value was 4 Mb. The genome coverage was 4% or more, which can cover microdeletion, microrepetition, and aneuploidy more than 4 Mb. The detailed procedures can be found in the previous study (Xiao *et al.*, 2021). Following biopsy, blastocysts were vitrified using the Kitazato vitrification system (Kitazato Corporation, Shizuoka, Japan) on Cryotop carriers and stored in liquid nitrogen. All experiments were carried out, and all data were analysed within the same local laboratory at JIAI.

The PGT-A reports categorize results as euploid, aneuploid, mosaic, or non-conclusive. These categories were defined based on mosaicism levels derived from intermediate copy number (ICN) analysis. Euploid was defined as a mosaicism level ≤20%, and corresponding blastocysts were recommended for priority transfer. Mosaic encompassed embryos with levels >20–80%, subdivided into low-level (>20–50%) and high-level (>50–80%) according to the degree of ICN deviation. Blastocysts with low-level mosaicism (20–50%) were retained and recommended for transfer after genetic counselling, whereas those with high-level mosaicism (>50%) were not recommended for transfer. Aneuploidy was assigned to embryos with a mosaicism level >80%, as well as to all cases of complex mosaicism (involving two or more chromosomes), based on ICN values reaching homozygous aneuploid cutoffs. Blastocysts for which a definitive diagnosis could not be established were reported as non-conclusive (Professional Committee on Reproductive Medicine, Chinese Medical Doctor Association (Professional Committee on Reproductive Medicine, Chinese Medical Doctor Association & Chinese Society of Reproduction Medicine, Chinese Medical Association, 2024).

### Frozen embryo transfer

Women in both groups underwent frozen embryo transfer at least 1 month after the stimulation cycle if they had at least one euploid blastocyst. Frozen embryo transfers were performed in natural cycles or hormone replacement cycles. Only euploid embryos were transferred, and only one blastocyst was replaced in each transfer. A pregnancy test was performed 2 weeks after the embryo transfer. Transvaginal ultrasonography was performed to confirm a gestational sac and a foetal heartbeat 2 weeks after a positive pregnancy test. Reproductive outcomes of the first frozen embryo transfer were recorded, namely, positive HCG level, clinical and ongoing pregnancy, multiple pregnancy, ectopic pregnancy, miscarriage, live birth, and birth weights of newborns.

### Outcome measures

The primary outcome of the study was the euploidy rate per woman. The secondary outcomes included number of mature oocytes, number of blastocysts, positive HCG level, clinical pregnancy, ongoing pregnancy, multiple pregnancy, miscarriage rate, ectopic pregnancy rate, live birth, and birth weight of newborns. Clinical pregnancy was defined as the presence of an intrauterine gestational sac by transvaginal ultrasound at 6 gestational weeks. Ongoing pregnancy was defined as a viable pregnancy beyond 12 weeks of gestation. Multiple pregnancy was diagnosed if there was more than one gestational sac detected on ultrasound at 6 weeks of pregnancy. Miscarriage was defined as clinically recognized pregnancy loss before 22 weeks of pregnancy, and the denominator for the miscarriage rate was the number of clinical pregnancies. A baby born alive after 22 weeks gestation was classified as a live birth.

### Sample size

According to our data from the centre, in a group of 284 women (mean age 36.9 years and SD 3.5 years) undergoing PGT-A for similar indications, the mean euploidy rate of blastocysts was 42.0% with SD of 34.0%. We hypothesized that with a difference in the euploidy rate of 10% between the PPOS versus GnRH antagonist groups, the sample size required would be 182 in each arm to give a power of 0.8 and a Type I error of 0.05. Considering a 10% dropout rate, an overall sample size of 400 women (200 in each group) was planned.

### Statistical analysis

All randomized participants were included in the intent-to-treat analysis. The primary outcome was euploidy rate per patient. For those with no blastocysts available for biopsy, the euploidy rate was assigned a value of zero. Normality of continuous variables was tested using the Kolmogorov–Smirnov test. Values are reported as the median (interquartile range) for non-normally distributed variables, the mean ± SD for normally distributed variables, or as numbers (percentages) for categorical variables. The differences in continuous variables between the PPOS and GnRH antagonist groups were analysed using the independent-sample Student’s *T*-test if the data followed normal distributions; otherwise, Mann–Whitney *U*-tests were applied. Categorical variables were analysed using the chi-squared test. All statistical analyses of the data were performed using SPSS v21.0 (IBM Corp., Armonk, NY, USA), with *P < 0.05 considered statistically significant.

## Results

### Study participants

Figure 1 presents a flowchart of the trial. Between June 2020 and August 2024, 400 women joined the study: 200 in the PPOS group and 200 in the antagonist group. The baseline characteristics of the participants were comparable between the two groups (Table 1).

**Figure 1.**
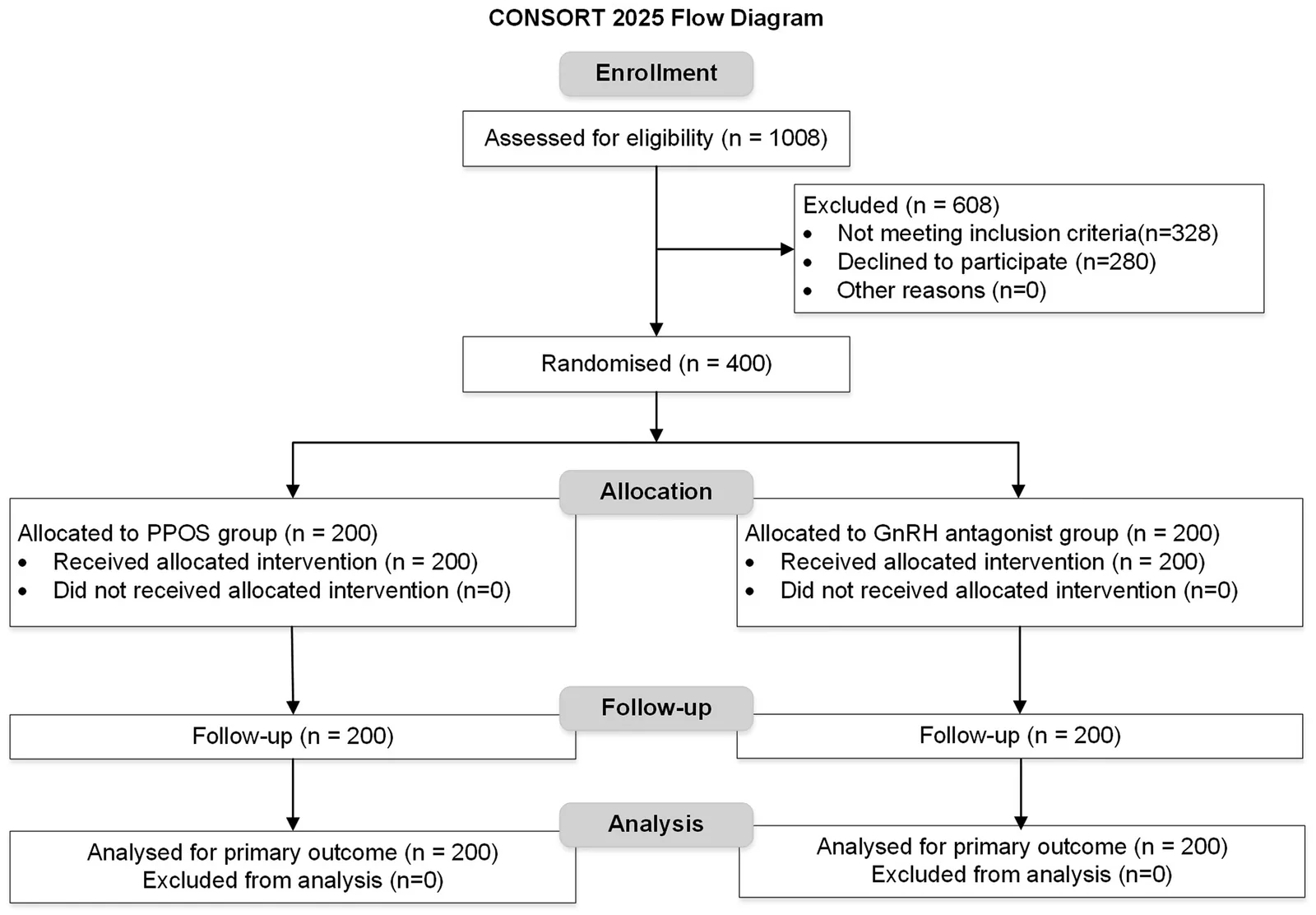
**Flowchart of the study.** This flowchart illustrates the enrolment, randomization, follow-up, and analysis of patients in a randomized controlled trial comparing the euploidy rate between the progestin-primed ovarian stimulation (PPOS) protocol and the GnRH antagonist protocol cycles with preimplantation genetic testing for aneuploidy.

**Table 1.** Demographic and clinical data of the IVF cycle.

	PPOS group	Antagonist group
	(n = 200)	(n = 200)
Female age (years)	34.0 (31.0–37.0)	34.0 (31.3–37.0)
Male age (years)	35.0 (32.0–39.0)	36.0 (33.0–39.0)
Body mass index of women (kg/m^2^)	21.6 (20.0–23.8)	21.9 (20.1–23.7)
Primary infertility, n (%)	28 (14.0)	24 (12.0)
Indication for PGT-A		
Recurrent pregnancy loss, n (%)	117 (58.5)	126 (63.0)
Repeated implantation failure, n (%)	38 (19.0)	34 (17.0)
Advanced maternal age, n (%)	26 (13.0)	24 (12.0)
Foetal aneuploidy in previous pregnancies, n (%)	16 (9.5)	16 (8.0)
Antral follicle count (n)	13 (9–18)	12 (9–18)
Basal serum FSH level (mIU/ml)	7.4 (6.5–8.7)	7.7 (6.6–8.9)

Values are presented as median (interquartile range), or n (%). The demographic and clinical data of the IVF cycle were comparable between the two groups. PPOS, progestin-primed ovarian stimulation; PGT-A, preimplantation genetic testing for aneuploidy.

### Outcomes of ovarian stimulation

The outcomes of ovarian stimulation are reported in Table 2. The PPOS group had a significantly lower total dose of gonadotrophin and significantly higher serum oestradiol and LH levels on the ovulation trigger day when compared with the antagonist group. No significant differences were observed between the two groups in the duration of stimulation, serum progesterone level on the trigger day, number of oocytes retrieved, number of mature oocytes, number of normally fertilized oocytes, number of blastocysts, number of cycles with no blastocyst for biopsy, and number of cycles with no euploid blastocysts for transfer.

**Table 2.** Characteristics and outcomes of ovarian stimulation.

	PPOS group	Antagonist group	*P* value
	(n = 200)	(n = 200)	
Total gonadotrophin dose (IU)	2400 (1669–3150)	2700 (2100–3300)	0.029*
Duration of stimulation (days)	10 (9–11)	10 (9–11)	0.13
Oestradiol level on trigger day (pg/ml)	3530 (2348–5523)	3145 (2218–4610)	0.044*
Progesterone level on trigger day (ng/ml)	1.2 (0.9–1.6)	1.1 (0.8–1.48)	0.057
LH level on trigger day (mIU/ml)	2.6 (1.7–4.3)	1.9 (1.2–2.9)	<0.001*
No. of oocytes (n)	11 (7–15)	10 (7–15)	0.936
No. of mature oocytes (n)	8.5 (6–12)	8.0 (5.2–13)	0.966
No. of fertilized oocytes (n)	7 (4.3–10.8)	7 (5–11)	0.966
No. of blastocysts (n)	3 (2–5)	3 (1–5)	0.949
No. of euploid blastocysts (n)	1 (1–2)	1 (1–2.5)	0.468
No. of cycles with no blastocyst for biopsy (%)	10 (5.0)	7 (3.5)	0.457
No. of cycles with no euploid blastocysts for transfer (%)	52 (26.0)	43 (21.5)	0.29
Euploidy rate per patient (%)	40.0 (0–66.7)	44.4 (25.0–71.4)	0.192
Aneuploidy rate per patient (%)	33.3 (0–65.3)	33.3 (0–65.0)	0.409
Mosaic blastocyst rate per patient (%)	0 (0–22.2)	0 (0–20.0)	0.746

Values are presented as median (interquartile range), or n (%). Continuous variables were analysed using the Mann–Whitney *U*-test. Categorical variables were analysed using the chi-squared test. *P < 0.05 was considered statistically significant. PPOS, progestin-primed ovarian stimulation.

The euploidy rate and the total number of euploid blastocysts per patient were comparable for the PPOS and antagonist groups (Table 2). In addition, the overall euploid blastocyst rate and the mosaicism rate, based on the total number of tested blastocysts, are presented in Supplementary Table S1.

### Pregnancy outcomes of the first frozen embryo transfer

A total of 288 women (136 and 152 women in the PPOS and GnRH antagonist groups, respectively) had their first frozen embryo transfer with one euploid blastocyst replaced. The clinical outcomes after the first frozen cycle, including the HCG positive rate, clinical pregnancy rate, ongoing pregnancy rate, live birth rate, ectopic pregnancy rate, and miscarriage rate, were all similar between the two groups (Table 3).

**Table 3.** Pregnancy outcomes of the first frozen embryo transfer cycle.

n (%)	PPOS group (n = 136)	Antagonist group (n = 152)	Relative risk (95% CI)	*P* value
HCG positive	91/136 (66.9)	99/152 (65.1)	0.924 (0.567–1.506)	0.750
Clinical pregnancy	77/136 (56.6)	80/152 (52.6)	1.175 (0.738–1.870)	0.498
Ongoing pregnancy	58/136 (42.6)	68/152 (44.7)	0.919 (0.576–1.465)	0.721
Ectopic pregnancy	2/77 (2.6)	1/80 (1.3)	2.133 (0.19–24.016)	0.530
Miscarriage	17/77 (22.1)	11/80 (13.8)	0.563 (0.244–1.295)	0.173
Live birth	58/136 (42.7)	68/152 (44.7)	1.089 (0.683–1.736)	0.721

Values are presented as n/N (%). Categorical variables were analysed using the chi-squared test. *P < 0.05 was considered statistically significant. PPOS, progestin-primed ovarian stimulation.

## Discussion

This randomized study demonstrated a similar euploidy rate of blastocysts in patients undergoing PGT-A and using the PPOS or GnRH antagonist protocols for ovarian stimulation. This finding is relevant for clinicians, since some controversies remain about the impact of progestins on oocyte quality and euploidy rates.

Many studies found that the PPOS protocol was non-inferior to the antagonist protocol in terms of the number of retrieved oocytes, number of embryos, and pregnancy rate. However, a randomized trial showed that the live birth rate was significantly lower in the PPOS protocol compared to the antagonist protocol, despite a comparable yield of mature oocytes in oocyte donation patients. The PPOS group had a biochemical pregnancy rate of 44.3% versus 56.2%, a clinical pregnancy rate of 29.9% versus 42.5%, and a live birth rate of 27.4% versus 36.9%, all of which were statistically significant (Beguería *et al.*, 2019). A limitation of this trial is that the oocyte recipients were not randomized.

Our study demonstrated that the number of euploid blastocysts and euploidy rate per woman were similar between the PPOS and GnRH antagonist protocols. These results are consistent with the results of previous published studies (La Marca *et al.*, 2020; Ata *et al.*, 2021; Yang *et al.*, 2022; Wang *et al.*, 2023, 2025; Vidal *et al.*, 2024), which showed that PPOS may not affect the embryo quality, at least when evaluated by analysing the chromosomes of the embryos. A recent randomized study reported that the euploidy rate, the number of blastocysts, and the number of euploid embryos were not significantly different in the patients receiving PPOS and the patients receiving GnRH antagonist. As expected, the live birth rate was also comparable as well (Wang *et al.*, 2025). In that randomized study, the sample size calculation was based on the euploidy rate in a group rather than per patient.

Our results indicated that progestins were capable of effectively preventing premature ovulation in PGT-A cycles; however, serum LH and oestradiol levels on the ovulation trigger day were significantly higher in the PPOS group when compared with the GnRH antagonist group. A potential explanation could be that the suppression effect of dydrogesterone on the pituitary may be weaker than the GnRH antagonist, despite being efficient enough to effectively control LH pulses. The numbers of oocytes obtained and fertilized oocytes were similar for the PPOS and antagonist groups.

We acknowledged some limitations of our study. One limitation was the relatively small sample size, as we aimed to detect a 10% difference in the euploidy rate per patient, and therefore a smaller difference cannot be excluded. Some women had no transferable blastocysts; thus, only a proportion of the women in both groups completed their first frozen embryo transfer cycle. Also, the pregnancy outcomes of first frozen embryo transfer cannot be considered the cumulative live birth rates and should not be used for direct protocol comparisons regarding clinical efficacy. Additionally, the study was not powered to detect differences in these secondary clinical endpoints, further underscoring their preliminary nature. Furthermore, the reported clinical pregnancy and live birth outcomes are derived solely from the transfer of embryos classified as euploid (≤20% abnormal cells). These results do not reflect the potential reproductive outcomes of transferring mosaic embryos and may not be generalizable to all patient populations undergoing PGT-A. Therefore, pregnancy outcomes should be interpreted with caution. Another limitation was the recruitment of women with indications for PGT-A only; hence, the results may not be extrapolated to the general population seeking IVF treatment. Additionally, our study evaluated the effect of dydrogesterone used in the PPOS protocol on the euploidy rate, and the effects of other progestins remain unknown.

In conclusion, the present randomized study demonstrated that the PPOS and GnRH antagonist protocols were associated with a similar euploidy rate in women undergoing PGT-A.

## Supplementary Material

hoag081_Supplementary_Data

## Data Availability

The data underlying this article will be shared on reasonable requests made to the corresponding author.

## References

[hoag081-B1] Alexandru P , Cekic SG, Yildiz S, Turkgeldi E, Ata B. Progestins versus GnRH analogues for pituitary suppression during ovarian stimulation for assisted reproductive technology: a systematic review and meta-analysis. Reprod Biomed Online 2020;40:894–903. 10.1016/j.rbmo.2020.01.027 32327297

[hoag081-B2] Ata B , Capuzzo M, Turkgeldi E, Yildiz S, La Marca A. Progestins for pituitary suppression during ovarian stimulation for ART: a comprehensive and systematic review including meta-analyses. Hum Reprod Update 2021;27:48–66. 10.1093/humupd/dmaa040 33016316

[hoag081-B3] Beguería R , García D, Vassena R, Rodríguez A. Medroxyprogesterone acetate versus ganirelix in oocyte donation: a randomized controlled trial. Hum Reprod 2019;34:872–880. 10.1093/humrep/dez034 30927417

[hoag081-B5] Cui L , Lin Y, Wang F, Chen C. Effectiveness of progesterone-primed ovarian stimulation in assisted reproductive technology: a systematic review and meta-analysis. Arch Gynecol Obstet 2021;303:615–630. 10.1007/s00404-020-05939-y PMC7960625 33433705

[hoag081-B7] Gardner DK , Lane M, Schoolcraft WB. Physiology and culture of the human blastocyst. J Reprod Immunol 2002;55:85–100. 10.1016/s0165-0378(01)00136-x 12062824

[hoag081-B8] Guan S , Feng Y, Huang Y, Huang J. Progestin-primed ovarian stimulation protocol for patients in assisted reproductive technology: a meta-analysis of randomized controlled trials. Front Endocrinol (Lausanne) 2021;12:702558. 10.3389/fendo.2021.702558 PMC8438422 34531825

[hoag081-B9] Huang B , Ren X, Wu L, Zhu L, Xu B, Li Y, Ai J, Jin L. Elevated progesterone levels on the day of oocyte maturation may affect top quality embryo IVF cycles. PLoS One 2016;11:e0145895. 10.1371/journal.pone.0145895 PMC4706317 26745711

[hoag081-B10] La Marca A , Capuzzo M, Sacchi S, Imbrogno MG, Spinella F, Varricchio MT, Minasi MG, Greco P, Fiorentino F, Greco E. Comparison of euploidy rates of blastocysts in women treated with progestins or GnRH antagonist to prevent the luteinizing hormone surge during ovarian stimulation. Hum Reprod 2020;35:1325–1331. 10.1093/humrep/deaa068 32395749

[hoag081-B11] Li H , Yu M, Zhang W, Chen J, Chen H, Lu X, Li L, Ng EHY, Sun X. Comparing blastocyst euploid rates between the progestin-primed and gonadotrophin-releasing hormone antagonist protocols in aneuploidy genetic testing: a randomised trial protocol. BMJ Open 2024;14:e079208. 10.1136/bmjopen-2023-079208 PMC10961518 38521533

[hoag081-B12] Massin N. New stimulation regimens: endogenous and exogenous progesterone use to block the LH surge during ovarian stimulation for IVF. Hum Reprod Update 2017;23:211–220. 10.1093/humupd/dmw047 28062551

[hoag081-B13] Melo MA , Meseguer M, Garrido N, Bosch E, Pellicer A, Remohí J. The significance of premature luteinization in an oocyte-donation programme. Hum Reprod 2006;21:1503–1507. 10.1093/humrep/dei474 16648153

[hoag081-B14] Pai AHY , Sung YJ, Li CJ, Lin CY, Chang CL. Progestin Primed Ovarian Stimulation (PPOS) protocol yields lower euploidy rate in older patients undergoing IVF. Reprod Biol Endocrinol 2023;21:72. 10.1186/s12958-023-01124-3 PMC10408156 37550681

[hoag081-B15] Pardiñas ML , Nohales M, Labarta E, De Los Santos JM, Mercader A, Remohí J, Bosch E, De Los Santos MJ. Elevated serum progesterone does not impact euploidy rates in PGT-A patients. J Assist Reprod Genet 2021;38:1819–1826. 10.1007/s10815-021-02197-y PMC8324650 34008094

[hoag081-B8128446] Professional Committee on Reproductive Medicine, Chinese Medical Doctor Association & Chinese Society of Reproduction Medicine, Chinese Medical Association, Chinese Expert consensus on genetic counseling and transfer strategies of mosaic embryos in PGT-A. Zhonghua Fu Chan Ke Za Zhi 2024;59:577–582. 10.3760/cma.j.cn112141-20240208-00088 39187404

[hoag081-B16] Racca A , De Munck N, Santos-Ribeiro S, Drakopoulos P, Errazuriz J, Galvao A, Popovic-Todorovic B, MacKens S, De Vos M, Verheyen G et al Do we need to measure progesterone in oocyte donation cycles? A retrospective analysis evaluating cumulative live birth rates and embryo quality. Hum Reprod 2020;35:167–174. 10.1093/humrep/dez238 31953546

[hoag081-B17] Racca A , Santos-Ribeiro S, Munck N, De Mackens S, Drakopoulos P, Camus M, Verheyen G, Tournaye H, Blockeel C. Impact of late-follicular phase elevated serum progesterone on cumulative live birth rates: is there a deleterious effect on embryo quality? Hum Reprod 2018;33:860–868. 10.1093/humrep/dey031 29481670

[hoag081-B18] Ubaldi F , Smitz J, Wisanto A, Joris H, Schiettecatte J, Derde MP, Borkham E, Van Steirteghem A, Devroey P. Oocyte and embryo quality as well as pregnancy rate in intracytoplasmic sperm injection are not affected by high follicular phase serum progesterone. Hum Reprod 1995;10:3091–3096. 10.1093/oxfordjournals.humrep.a135864 8822420

[hoag081-B19] Vanni VS , Somigliana E, Reschini M, Pagliardini L, Marotta E, Faulisi S, Paffoni A, Vigano' P, Vegetti W, Candiani M, Papaleo E. Top quality blastocyst formation rates in relation to progesterone levels on the day of oocyte maturation in GnRH antagonist IVF/ ICSI cycles. PLoS One 2017;12:e0176482. 10.1371/journal.pone.0176482 PMC5435161 28520729

[hoag081-B20] Vidal MDM , Martínez F, Rodríguez I, Polyzos NP. Ovarian response and embryo ploidy following oral micronized progesterone-primed ovarian stimulation versus GnRH antagonist protocol. A prospective study with repeated ovarian stimulation cycles. Hum Reprod 2024;39:1098–1104. 10.1093/humrep/deae047 38498835

[hoag081-B21] Wang L , Wang J, Zhang Y, Qian C, Wang X, Bai J, Li F, Chen Z, Ai A. Analysis of euploidy rates in preimplantation genetic testing for aneuploidy cycles with progestin-primed versus GnRH agonist/antagonist protocol. Eur J Med Res 2023;28:28. 10.1186/s40001-023-01000-1 PMC9841681 36642707

[hoag081-B22] Wang L , Wang JY, Zhang Y, Qian C, Wang XH, Ng EHY, Ai A, Chen ZQ. Comparison of the euploidy rate in preimplantation genetic testing for aneuploidy cycles following progestin-primed versus gonadotropin-releasing hormone antagonist protocol: a randomized controlled study. Reprod Biol Endocrinol 2025;23:67. 10.1186/s12958-025-01404-0 PMC12070503 40361159

[hoag081-B23] Xiao M , Lei CX, Xi YP, Lu YL, Wu JP, Li XY, Zhang S, Zhu SJ, Zhou J, Li X et al Next-generation sequencing is more efficient at detecting mosaic embryos and improving pregnancy outcomes than single-nucleotide polymorphism array analysis. J Mol Diagn 2021;23:710–718. 10.1016/j.jmoldx.2021.02.011 33781963

[hoag081-B24] Xu B , Li Z, Zhang H, Jin L, Li Y, Ai J, Zhu G. Serum progesterone level effects on the outcome of in vitro fertilization in patients with different ovarian response: an analysis of more than 10,000 cycles. Fertil Steril 2012;97:1321–1327. 10.1016/j.fertnstert.2012.03.014 22494924

[hoag081-B25] Yang L , Luo K, Lu G, Lin G, Gong F. Euploidy rates among preimplantation genetic testing for aneuploidy cycles with oral dydrogesterone primed ovarian stimulation or GnRH antagonist protocol. Reprod Biomed Online 2022;45:721–726. 10.1016/j.rbmo.2022.03.003 35989167

[hoag081-B26] Zolfaroli I , Ferriol GA, Mora JJH, Cano A. Impact of progestin ovarian stimulation on newborn outcomes: a meta-analysis. J Assist Reprod Genet 2020;37:1203–1212. 10.1007/s10815-020-01755-0 PMC7244671 32215824

