# Supplementary material for: Comparing the euploidy rate between the progestin-primed and gonadotrophin-releasing hormone antagonist protocols in preimplantation genetic testing for aneuploidy: a randomized controlled trial
Source: Hum Reprod Open. 2026 Sep 15;2026(4):hoag081. doi: 10.1093/hropen/hoag081 (PMC13626714; doi:10.1093/hropen/hoag081)
Supplement: Supplementary file 1 [file hoag081_supplementary_data.docx]

**Supplementary Table S1 Embryo outcomes per total blastocysts following ovarian stimulation and PGT‑A**

|  | PPOS Group | Antagonist Group |
| --- | --- | --- |
|  | (n=200) | (n=200) |
| Euploid blastocyst rate (%, /biopsied blastocyst number) | 44.5 (302/679) | 49.4 (332/672) |
| Aneuploid blastocyst rate (%, /biopsied blastocyst number) | 40.6 (276/679) | 36.6 (246/672) |
| Mosaic blastocyst rate (%, /biopsied blastocyst number) | 14.3 (97/679) | 13.2 (89/672) |
| Non-conclusive rate (%, /biopsied blastocyst number) | 0.6 (4/679) | 0.7 (5/672) |

Values are presented as % (n/N), where n = number of events and N = total biopsied blastocyst number in each group. PPOS, progestin-primed ovarian stimulation.
